# Breast carcinoma metastasis and Wolf’s isotopic response^⋆^

**DOI:** 10.1016/j.abd.2021.10.004

**Published:** 2022-06-05

**Authors:** Bruna Rocha Reolão, Diego Salomón Mora, Michele Caroline dos Santos Garcia, Renan Rangel Bonamigo

**Affiliations:** a Santa Casa de Misericórdia de Porto Alegre, Porto Alegre, RS, Brazil; b Ambulatório de Dermatologia Sanitária do Rio Grande do Sul, Porto Alegre, RS, Brazil; c Faculty of Medicine, Universidade Federal do Rio Grande do Sul, Porto Alegre, RS, Brazil

**Keywords:** Breast neoplasms, Herpes zoster, Neoplasm metastasis, Skin neoplasms, Varicella-Zoster virus infection

## Abstract

Wolf’s isotopic phenomenon occurs when a new dermatosis appears on a site that has already healed from a previous dermatological disease of another etiology. This report describes the case of a 44-year-old female patient undergoing treatment for breast carcinoma who recently had brownish erythematous lesions appearing on the scar region of previous herpes zoster on the right hemithorax. Histopathology and immunohistochemistry examination confirmed skin metastasis of breast cancer. Herpes zoster scars require attention due to the possibility of an isotopic response as a facilitating factor in some dermatoses, sometimes severe ones, such as neoplasms.

## Introduction

Wolf’s isotopic response (WIR) is defined as the occurrence of a new cutaneous dermatosis that appears on the site of a previous, healed, dermatological disease. Many skin lesions in the topography of herpes zoster (HZ) have been described as WIR; this viral infection is the most commonly described primary disease predisposing to this response, and the term “postherpetic Wolf’s isotopic response” has been suggested by several authors.^1^ Breast carcinoma is unusually reported as being related to WIR, and the histopathological study is critical for the final diagnosis.^1^, ^2^

## Case report

This report describes a 44-year-old woman undergoing treatment for metastatic breast cancer to the lung, pleura and bones, which was submitted to a radical mastectomy, chemotherapy with docetaxel, and previous radiotherapy, and is currently undergoing chemotherapy with fulvestrant and ribociclib. She had a history of HZ on the right hemithorax for two months, with clinical diagnosis attained by the initial classic presentation, good therapeutic response to acyclovir, and complete remission of the condition. She had started one week before with new skin lesions on the same site but with slightly different characteristics, such as slight lesion infiltration and absence of vesicles, erosions or secretion.

**Figure 1:**
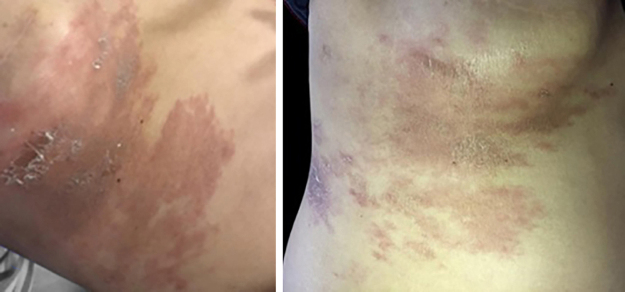
Brownish erythematous, desquamative plaque on the right hemithorax, with a diagnosis of metastatic breast carcinoma: topography of post-herpetic WIR.

**Figure 2:**
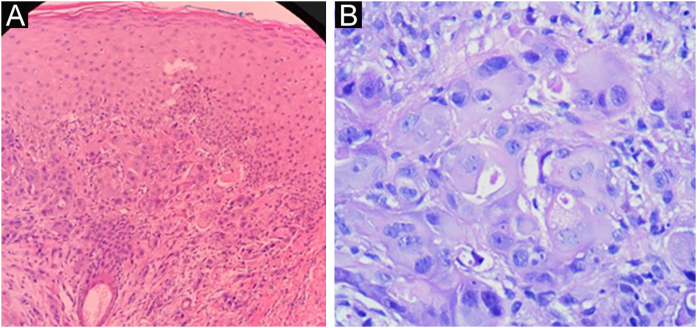
Histopathology of the right hemithorax plaque. A. Poorly differentiated carcinoma on the left, (Hematoxylin & eosin, ×100); B. on the right (Hematoxylin & eosin, ×400).

**Figure 3:**
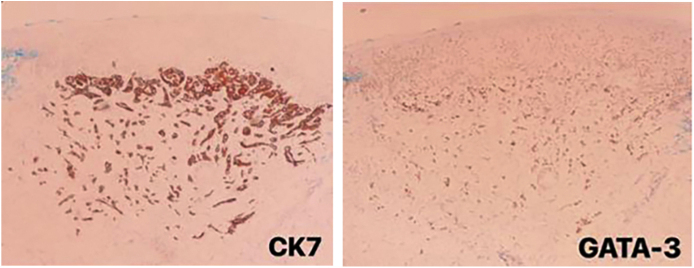
Immunohistochemical panel for metastatic breast carcinoma: positive for CK7 and GATA-3 markers.

On physical examination, the right hemithorax showed some macules and an extensive brownish erythematous plaque, with little infiltration and mild desquamation (Fig. 1). Histopathological examination of the skin plaque showed metastasis of poorly differentiated carcinoma, and immunohistochemistry revealed positivity for CK-7 and GATA-3, confirming cutaneous metastasis of poorly differentiated breast carcinoma, and positivity for Ki-67 in 20% of the neoplastic cells (Figure 2, Figure 3).

## Discussion

In the present case, in the context of immunosuppression (baseline neoplasm under chemotherapy), the initial diagnosis of another episode of herpes zoster would be possible, but the differential diagnosis with local tumor infiltration would also need to be considered when recalling the presence of WIR, particularly due to the atypical configuration of the disease and the prolonged clinical picture.^1^, ^2^, ^3^

WIR is a rare, probably underdiagnosed entity and consists of the development of a dermatosis on the site of a previous skin lesion, of different etiology and already healed. In most cases, the first dermatosis is herpes (zoster or simplex), although other skin lesions can also precede the second lesion. The isotopic response can reveal the existence of several conditions – such as granulomatous diseases, impetigo, psoriasis, and even malignant tumors.^4^

The term “Wolf’s isotopic phenomenon” was introduced in 1995. Although the first described clinical case dates from 1929, the understanding of this phenomenon is recent and the number of known cases is relatively scarce.^4^, ^5^, ^6^ This response differs from Köebner isomorphic phenomenon, as it is characterized by the development of the same disease on another skin site, induced by traumatic and/or inflammatory processes, whereas WIR is characterized by the appearance of a new disease on the same previously affected site.^7^

The pathogenesis of WIR remains unclear and includes vascular, immunological, and viral factors. Some authors state that the herpetic infection, by destroying cutaneous nerve fibers, could trigger immune dysregulation phenomena, thus favoring the development of inflammatory reactions or causing local immunosuppression (important in cases of tumor infiltration).^1^, ^2^, ^3^

The differential diagnosis between the two dermatoses is extremely important since the treatment differs between them. HZ is a cutaneous manifestation resulting from the reactivation of a previous infection by the Varicela-zoster virus, which remains latent in the sensory ganglia, and typically presents with a prodromal phase of nonspecific symptoms and subsequently with the characteristic erythematous-papulovesicular rash, which follows the path of a dermatome, generally respecting the midline.

Histopathological examination shows intraepidermal vesicles or ulceration, as well as epidermal necrosis and ballooning ‒ large, pale keratinocytes with metallic gray nuclei, chromatin margination at the edge of the nucleus, sometimes with pink intranuclear inclusions surrounded by an artefactual cleft (Cowdry’s type A inclusions, Lipschutz bodies, “owl eyes”), acantholysis, or multinucleated keratinocytes. There may also be extravasated erythrocytes and diffuse perivascular lymphocytes or neutrophils, sometimes with leukocytoclastic vasculitis. Other diagnostic methods available for HZ are cultures, fluorescent antibody smear techniques, or Tzanck preparations.^8^

Breast carcinoma is the neoplasm that most often results in cutaneous metastasis in women, usually detected on the trunk as an indurated/infiltrated plaque or nodule, which is commonly an adenocarcinoma. Breast cancer metastasis usually occurs within the first three years after the neoplasm diagnosis and in women aged between 50 and 70 years. Histopathological examination shows tumor nodules with pleomorphic and hyperchromatic nuclei, with increased mitosis in the dermis. In some cases, glandular structures may contain mucin.

Most metastatic breast carcinomas express, on immunohistochemistry, CK7 and CK19, estrogen and progesterone receptors, mammaglobin, GCDFP-15, CEA, and E-cadherin, but are negative for CK20, CK5/6, CD10 and TTF-1.^8^, ^9^ GATA-3 marker has high sensitivity and specificity for the diagnosis of breast carcinoma, especially for the estrogen-receptor-positive and Her2 positive subtype. Ki67, on the other hand, is a biomarker widely used to measure and monitor the cell proliferation rate in tumors, including breast carcinomas, with very high or very low rates being considered the most useful in prognostic assessment.^10^

In the present clinical case, histopathology showed characteristics of a poorly differentiated carcinoma metastasis, and immunohistochemistry was crucial, confirming the hypothesis of breast carcinoma metastasis, as it expressed the CK-7 and GATA-3 markers, in addition to Ki-67 in 20% of the neoplastic cells.

## Conclusion

Varicella-Zoster virus infection results in increased chances of developing HZ and WIP. It is very important to pay attention to the possibility of the emergence of different dermatoses in dermatomes that were previously affected by the varicella-zoster virus, including metastatic neoplasms, as in the case described herein. In cases of suspected WIR, histopathological and immunohistochemical studies are extremely relevant.

## Financial support

None declared.

## Authors’ contributions

Bruna Rocha Reolão: Approval of the final version of the manuscript; design and planning of the study; drafting and editing of the manuscript; intellectual participation in the propaedeutic and/or therapeutic conduct of the studied cases; critical review of the literature; critical review of the manuscript.

Diego Salomón Mora: Approval of the final version of the manuscript; design and planning of the study; drafting and editing of the manuscript; intellectual participation in the propaedeutic and/or therapeutic conduct of the studied cases; critical review of the literature; critical review of the manuscript.

Michele Caroline dos Santos Garcia: Approval of the final version of the manuscript; design and planning of the study; drafting and editing of the manuscript; intellectual participation in the propaedeutic and/or therapeutic conduct of the studied cases; critical review of the literature; critical review of the manuscript.

Renan Rangel Bonamigo: Approval of the final version of the manuscript; design and planning of the study; effective participation in research orientation; intellectual participation in the propaedeutic and/or therapeutic conduct of the studied cases; critical review of the manuscript.

## Conflicts of interest

None declared.
